# Combination therapy in combating cancer

**DOI:** 10.18632/oncotarget.16723

**Published:** 2017-03-30

**Authors:** Reza Bayat Mokhtari, Tina S. Homayouni, Narges Baluch, Evgeniya Morgatskaya, Sushil Kumar, Bikul Das, Herman Yeger

**Affiliations:** ^1^ Developmental and Stem Cell Biology, The Hospital for Sick Children, Toronto, Ontario, Canada; ^2^ Department of Paediatric Laboratory Medicine, The Hospital for Sick Children and Institute of Medical Science, University of Toronto, Toronto, Ontario, Canada; ^3^ Department of Pathology and Molecular Medicine, Queen’s University, Kingston, Ontario, Canada; ^4^ Department of Immunology and Infectious Diseases, The Forsyth Institute, Cambridge, Massachusetts, USA

**Keywords:** Nrf2-Keap1, HIF-1alpha, carbonic anhydrase 9 (CAIX), histone deacetylase inhibitor (HDACi), carbonic anhydrase inhibitor (CAI)

## Abstract

Combination therapy, a treatment modality that combines two or more therapeutic agents, is a cornerstone of cancer therapy. The amalgamation of anti-cancer drugs enhances efficacy compared to the mono-therapy approach because it targets key pathways in a characteristically synergistic or an additive manner. This approach potentially reduces drug resistance, while simultaneously providing therapeutic anti-cancer benefits, such as reducing tumour growth and metastatic potential, arresting mitotically active cells, reducing cancer stem cell populations, and inducing apoptosis. The 5-year survival rates for most metastatic cancers are still quite low, and the process of developing a new anti-cancer drug is costly and extremely time-consuming. Therefore, new strategies that target the survival pathways that provide efficient and effective results at an affordable cost are being considered. One such approach incorporates repurposing therapeutic agents initially used for the treatment of different diseases other than cancer. This approach is effective primarily when the FDA-approved agent targets similar pathways found in cancer. Because one of the drugs used in combination therapy is already FDA-approved, overall costs of combination therapy research are reduced. This increases cost efficiency of therapy, thereby benefiting the “medically underserved”. In addition, an approach that combines repurposed pharmaceutical agents with other therapeutics has shown promising results in mitigating tumour burden. In this systematic review, we discuss important pathways commonly targeted in cancer therapy. Furthermore, we also review important repurposed or primary anti-cancer agents that have gained popularity in clinical trials and research since 2012.

## SYSTEMATIC REVIEW: COMBINATION THERAPY IN COMBATING CANCER

## BACKGROUND

According to the American Cancer Society's global cancer facts report, the average 5-year survival rates for common pediatric and adult cancer subtypes in the North America are 65-95% and 14-56% respectively, which are still quite low [1, 2]. Cancer remains the leading cause of childhood deaths in the United States, and the economic and financial burden for cancer research is increasing [3]. Age-standard cancer mortality rates for all types of malignancies have decreased, but newer drugs only contribute to a small percentage of this improvement [4]. This is primarily because the development of new pharmaceutical anti-cancer agents is laborious and expensive, requiring initial *in vitro* and *in vivo* experimentation, and subsequent clinical trials before receiving FDA approval. It is estimated that a newly designed drug takes 15 years to enter the pharmaceutical market [5]. Consequently, it is important to find more efficient methodical approaches that are also economically feasible. Newer approaches that do not rely solely on a single agent's traditional cytotoxicity profile are required in order to provide a more targeted, efficient and enhanced form of cancer therapy. For instance, monoclonal antibodies and chemoprevention with naturally- compounds are examples of new strategies to prevent or treat cancer [6–9].

The combination of two or more therapeutic treatments to specifically target cancer-inducing or cell-sustaining pathways is a cornerstone of cancer therapy [10, 11]. Although the mono-therapy approach is still a very common treatment modality for many different forms of cancer, this conventional method is generally deemed less effective than the combination therapy approach [SOURCE]. Conventional mono-therapeutic techniques non-selectively target actively proliferating cells, which ultimately leads to the destruction of both healthy and cancerous cells. Chemotherapy can be toxic to the patient with multiple side effects and risks, and can also strongly reduce their immune system by affecting bone marrow cells and increasing susceptibility to host diseases [12, 13]. Although combination therapy can be toxic if one of the agents used is chemotherapeutic, the toxicity is significantly less because different pathways will be targeted. Ultimately, this works in a synergistic or additive manner, and therefore a lower therapeutic dosage of each individual drug is required [14, 15]. Additionally, combination therapy may be able to prevent the toxic effects on normal cells while simultaneously producing cytotoxic effects on cancer cells. This may occur if one drug in the combination regimen is antagonistic, in terms of cytotoxicity, to another drug in normal cells, essentially protecting normal cells from cytotoxic effects [16]. Consider a combination regimen that includes a caspase-inhibitor, such as Z-DEVD-fmk, and an apoptosis-inducing agent. Specifically, Z-DEVD-fmk-resistant cancer cells that express p-glycoprotein will pump out Z-DEVD-fmk, but the apoptosis-inducing agent would induce apoptosis in cancer cells [16]. However, most normal cells do not express p-glycoprotein and thus they will be directly affected by Z-DEVD-fmk and apoptosis will resume [16]. This method would essentially increase the therapeutic index of cancer therapy and produce a more potent cytotoxic effect [16]. Further, monotherapy treatment is more susceptible to drug resistance because the constant treatment with a single compound induces cancer cells to recruit alternative salvage pathways [17, 18]. As an example, cells in adenocarcinoma, when treated with doxorubicin, upregulate an ATP-dependent cassette pump to eliminate the drug, leading to a state of drug resistance [17]. However, combination therapy can produce a more effective treatment response in fewer cycles, and therefore this treatment modality reduces the incidence of resistance [19, 20]. Finally, chemotherapeutics essentially does not eliminate cancer stem cells (CSCs) successfully due to their non-selective therapeutic approach. This is a major disadvantage because neoplasms harbour a subpopulation of CSCs that give the tumour its self-renewal, differentiation and invasive potential [21]. However, combination therapy that includes agents that target CSCs (examples are the notch inhibitor and the gamma-secretase inhibitor) would therefore reduce drug resistance and attenuate the likelihood of relapse [22].

Combination therapy was first conceptualized in 1965, where Emil Frei, James F. Holland and Emil J. Freireich postulated the possibility of the first ever combination chemotherapy for acute leukemia [23]. Pediatric patients with acute lymphocytic leukemia were treated with the combination of methotrexate, 6-mercaptopurine, vincristine and prednisone (formally known as the POMP regimen) and was proven successful in reducing tumour burden and prolonging remission [23]. As a consequence of the success of the POMP regimen, research in cancer therapy became focused on investigating combination therapies that target different pathways to create a synergistic or additive effect. For example, in a study by Quinn *et al*., it was discovered that sabutoclax, a pan-Bcl-2 inhibitor, in combination with minocycline, an antibiotic that has previously displayed anti-cancer effects acted synergistically on the intrinsic apoptotic pathway. In addition, this combination displayed selective toxicity and a reduction in tumour growth *in vitro* and *in vivo* on pancreatic ductal adenocarcinoma [24]. Thus, using a combination of compounds that target different pathways, a synergistic or potentiation effect could yield significant anti-cancer results.

Recently, a newer approach in cancer therapy that focuses on restrictive combinations (RC) of drugs is emerging, although this approach has not yet been tested on human patients. This restrictive approach focuses on strategic dosing and drug administration in order to spare normal cells while simultaneously creating a targeted cytotoxic effect on cancer cells. To achieve this, the combination regimen takes advantage of the slight differences between cancer cells and normal cells, such as the absence of a target (lack of p53), or by the presence of a target (surface marker) [25]. The type of drugs used in the combination regimen and the sequence in which they are administered depends on what the slight differences are between cancerous tissue and the normal cells [25]. For example, a p53-inducing agent, such as low dose doxorubicin, causes G1/G2 arrest in non-target cells [25]. This arrest only occurs in normal cells if the cancerous cells lack p53. This arrest further protects normal cells from the cytotoxicity of the following drug that is intended to specifically kill cancer cells. In the next step of the combination regimen, taxol is added to induce mitotic arrest in actively dividing cells, targeted towards cancer cells [25]. Additional drugs can be added to increase the therapeutic potency in a synergistic manner but without undesirable side-effects [25]. Because cancer cells are heterogeneous, this method of treatment is alternated with standard therapy in order to eliminate all cancer cells. As a result, RC limits treatment resistance, lowers toxicity, and increases efficacy [25].

Another therapeutic approach that has gained publicity in cancer research is “drug repositioning”. Drug re-purposing is a therapeutic approach where current pharmaceutical agents primarily used for non-cancerous diseases are being used for cancer treatment [26]. This approach is efficient because the FDA-approved drug would have already passed drug safety protocols and has a known pharmacokinetic profile [27]. One such example of drug re-positioning is acetazolamide, (AZ; Diamox), a pan-carbonic anhydrase inhibitor. AZ is typically used for the treatment of glaucoma, epilepsy, and altitude sickness, but has recently been tailored for the treatment of cancer [15, 28]. Its cancer-targeting relevance came to the attention of researchers when it was discovered that cancer cells display high carbonic anhydrase activity, which correlates with malignant behavior; therefore, inhibiting this activity would create anti-cancer effects. In another example, Rapalog, an immunosuppressant that is primarily used after organ transplantation to prevent graft rejection, has shown to have cancer prevention properties [29]. One possible explanation for Rapalog's cancer preventative activity revolves around its mTOR inhibitory role [29]. Since mTOR activity is high in malignant cancerous tissue, Rapalog counteracts mTOR activity, preventing the formation of tumours [29].

Drug repositioning has also shown to be useful when traditional anti-cancer monotherapy has failed to provide a safe and tolerable treatment for cancer patients. For instance, the combination of drugs may consist of a repurposed neo-protector agent, such as a cytostatic agent that protects normal cells by arresting cell growth, and a secondary or tertiary agent that kills cancer cells [30]. The neo-protector may be a repurposed agent that was normally intended either as an anticancer drug or another disease-related therapeutic, but has shown to also display neo-protective roles over normal cells in cancer therapy [30]. In this way, drug repositioning has provided cancer research with new insights into efficiency and efficacy, while also reducing the financial burden associated with novel drug discovery. All in all, it would be reasonable to suggest that more adequately well-designed clinical research studies that test the combination of a repurposed therapeutic agent and another cytotoxic agent should be done in order to achieve greater efficacy in an expedient manner.

In this review, we discuss some important cellular pathways in cancer whose investigations have produced copious results in the PubMed database search engine. Pathways involved in the induction and sustaining of cancer that can be targeted include autocrine growth factors, hypoxia, carbonic anhydrase, antioxidant response, apoptosis, angiogenesis and epigenetic factors. These pathways have strong implications in cancer, and our current investigations, in particular, have further supported their roles in tumorigenesis [15, 28, 31]. Here, we provide an updated clinical review of the most current repurposed and prospective therapeutic combination treatments that target these pathways.

## PATHWAYS INVOLVED IN TUMOUR GROWTH

### 

#### Regulation of anti-oxidant response and tumorigenesis through Nrf2 activation

Under extreme oxidative intrinsic and extrinsic stress, cells have developed a systematic pathway to cope with the stress through an antioxidant response, namely the Nrf2-Keap1 pathway. Under quiescent conditions, Kelch-like ECH-associated protein-1 (Keap1), an electrophile and oxidant sensor, functions as an adaptor of Cul3-based E3 ligase and subsequently promotes Nrf2 degradation. Specifically, Keap1 first homodimerizes at the BTB domain, and the BTB domain targets the Neh2 region of nuclear factor erythroid 2-related factor 2 (Nrf2), a basic leucine zipper transcription factor (bZIP) to the E3 ligase complex. Once this interaction occurs, Nrf2 becomes ubiquitinated, targeting it for proteasomal degradation [32, 33]. However, under extreme oxidative stress, either due to the build-up of carcinogens or reactive oxygen species (ROS), Nrf2 is actively localized in the nucleus to elicit an anti-oxidative response. Nrf2 is also involved in regulating drug metabolism, cytoprotection, proliferation, growth differentiation and apoptosis [33]. Moreover, the Nrf2 antioxidant response regulates tumorigenicity in two ways [32]. Firstly, Nrf2 regulation occurs through Keap1-dependent and Keap1-independent mechanisms. In the Keap1-dependent mechanism, when intracellular ROS is elevated, the cysteine residues of Keap1 become oxidized, enabling Keap1 to dissociate from Nrf2 [34]. The sulfhydryl groups of cysteine residues are involved in sensing redox and electrophilic changes in the local environment by covalently binding to electrophiles [35]. Specifically, the cysteine residues (Cys273 and Cys288) in the intervening linker region of Keap1 are most reactive and sensitive to oxidative changes [36]. This dissociation of Keap1 from Nrf2 leads to the translocation of Nrf2 to the nucleus [34]. The Keap1-independent mechanisms of Nrf2 regulation primarily involve xenobiotic interactions (i.e. by; sulforaphane), Nrf2 transcriptional regulation and autoregulation, and post-transcriptional regulation, such as through microRNAs, phosphorylation and acetylation [33]. Once Nrf2 translocates to the nucleus, Nrf2 recruits other transcriptional machinery, such as CREB binding protein (CBP), coactivator-associated arginine methyltransferase (CARM1) and protein arginine methyl-transferase (PRMT1), leading to transactivation [33]. Further, Nrf2 heterodimerizes with either MAF (proto-oncogene c-Maf), activating transcription factor (ATF) or AP-1 (activator protein-1 transcription factor) and subsequently binds to a specific promoter region (a *cis*-acting enhancer sequence (TCAG/CXXXGC), termed the antioxidant response element (ARE) element of antioxidant genes [33, 37, 38]. Phase 2 detoxifying enzymes, NAD(P)H quinone reductase 1 (NQO1), glutathione S-transferases (GSTs), heme oxygenase 1, thioredoxin reductase, and keto reductases are primarily involved in detoxifying carcinogens [34, 39, 40]. In addition, other enzymes such as superoxide dismutase (SOD), catalase, and glutathione peroxidases are also regulated by the ARE transcriptional region [41]. Moreover, evidence of Nrf2-dependent chemopreventive activity was observed by Iida *et al*., where Nrf2 double-knockout C57BL/6 mice were more susceptible to the carcinogenic effects of a urinary bladder-specific carcinogen, *N*-nitrosobutyl (4-hydroxybutyl) amine (BBN), even when oltipraz, an inducer of phase 2 detoxifying enzymes genes that has a carcinogenic preventive role, was added [42].

Furthermore, the second way Nrf2 regulates tumorigenicity is by promoting the growth and survival of cancer cells that have been already initiated because Nrf2 and its antioxidant response participate in helping the tumour deal with oxidative stress [43]. Nrf2 promotes cancer cell proliferation since activation of phase 2 enzymes helps to reduce reactive oxygen species that would normally lead to apoptosis. For example, Nrf2 was upregulated in hepatocellular carcinoma, and in the same study it was shown that Nrf2 positively regulates GSTP1 (an enzyme that has a role in detoxification) [38]. These results indicate how upregulation of Nrf2 is a positive indicator of tumorigenesis due to its ability to help cancer cells cope with oxidative stress.

### Combination of pharmaceutical agents targeting antioxidant response pathways

Due to the role of Nrf2 in tumour prevention and progression, there are some pharmaceutical agents that induce the upregulation of Nrf2 expression. One such pharmaceutical agent is curcumin, the principal curcuminoid derived compound from turmeric (*Curcuma longa*) in the ginger family, Zingiberaceae [44]*.* Curcumin suppresses the activity of carcinogens in a Nrf2-dependent manner [45]. Curcuminoids are linear diarylheptanoids that upregulate Nrf2 expression and induce Nrf2 translocation to the nucleus to elicit its antioxidant effects by stabilizing protein levels of Nrf2. In addition, curcuminoids upregulate glutathione levels which have been shown to reduce ROS levels and remove carcinogens, aiding in chemoprevention [46]. In a phase II double-blind randomized study, curcuminoids in combination with chemotherapy have displayed enhanced efficacy with regards to reduced adverse side effects and improved quality of life in patients with solid tumors, such as colorectal, gastric, and breast cancer [47]. Curcuminoids in combination with chemotherapy have demonstrated an overall positive outcome, and have also shown to increase the survival rate in some patients [48, 49].

Resveratrol, a phytoalexin derived from plants, has displayed anti-oxidative and chemopreventive effects through the activation of Nrf2 and consequently GSH expression [45].

Our studies, as well as others, have shown the effectiveness of resveratrol in combination therapy *in vitro* and *in vivo* [50–53]. Chemoprevention is difficult to assess clinically, however, which contributes to the lack of phase II and III trials. However, a phase I trial has shown that micronized resveratrol (SRT501) is a good drug candidate for metastatic prevention. In this study, upregulation of caspase-3, an apoptotic marker, occurred in 39% of patients with hepatic malignancies; this demonstrates the effectiveness of resveratrol in preventing malignancies in other organs [54]. Another agent, squalene, a natural isoprenoid, has also displayed chemopreventive properties. Our lab showed that this antioxidant compound has cytoprotective properties against the side effects of chemotherapy. Squalene reduces ROS levels and upregulates glutathione levels among other detoxifying enzymes, with no effect on tumor cells as in neuroblastoma, small cell carcinoma and medulloblastoma xenografts [55]. Squalene appears to protect against chemotherapy toxicity and might be a potent adjunct to anti-cancer treatments.

**Figure 1:**
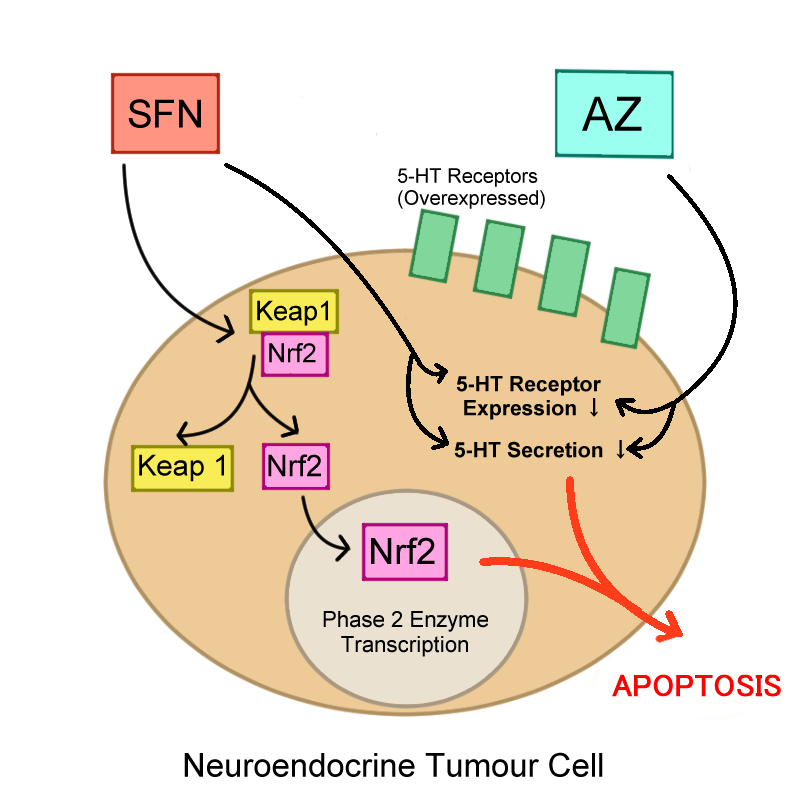
5-HT and Nrf2

Naturally derived, sulforaphane (SFN) has gathered a lot of attention in cancer chemoprevention. SFN, an isothiocyanate in the family of organosulfur compounds, has chemopreventive properties that are thought to be due to potent upregulation of Nrf2. Under normal cellular conditions, the transcriptional activity of Nrf2 is regulated by the interaction of Nrf2 with the Keap1 dimer; this interaction creates a Nrf2-Keap1 complex and prevents Nrf2 from translocating into the nucleus [40]. SFN has been shown to disrupt the Nrf2-Keap1 complex by targeting and modifying the C151 domain of Keap1 and thus diminishing the interaction of Nrf2 with Keap1 [40]. When Nrf2 is not bound with Keap1, it is free to translocate to the nucleus and elicit its antioxidant effects by upregulating Phase 2 enzymes (Figure 1) [40]. SFN, however, is theorized to also take on an inhibitory role when combined with other cytotoxic drugs for the treatment of cancer. To test this theory, Kallifatidis *et al.,* combined SFN with taxol in treatment of prostate cancer cell line DU145, and observed that SFN potentiated the effects of low doses of taxol; the combination inhibited viability and the clonogenicity of this cell line [56]. Further, SFN has demonstrated a significant ability to inhibit proliferation, induce apoptosis and induce G^2^/M cell cycle arrest *in vitro* and *in vivo* in multiple myeloma cells and myeloma xenograft model, respectively [57]. Although SFN showed efficacy in targeting myeloma cells, SFN also enhances the cytotoxicity of other anti-myeloma drugs when used in a combination regimen. SFN positively enhanced bortezomib, lenalidomide, and conventional drugs, such as dexamethasone, doxorubicin, and melphalan in a synergistic manner [57]. Likewise, SFN also potentiated the apoptotic effects of oxaliplatin in colorectal cancer Caco-2 cells [58]. A similar pattern was observed in our lab, where the combination of SFN and AZ showed enhanced efficacy in reducing tumor growth and clonogenic ability in bronchial carcinoid cell lines H727 and H720 (Figure 1) [15] and bladder cell lines HTB-9 and RT112(H) [28]. It is, therefore, reasonable to argue that the use of combination therapeutics that includes an isothiocyanate, specifically SFN, may show enhanced therapeutic effects. So far, SFN has displayed promising results in *in vitro* and *in vivo* studies but has not yet been tested in clinical trials, although its efficacy in combination therapy warrants further studies. Moreover, many cancer cells with elevated levels of Nrf2 have shown enhanced growth potential, and increased drug resistance. One such example is seen in human lung cancer, where elevated Nrf2 expression leads to cisplatin resistance [59]. Thus, it can be argued that a therapeutic agent that reduces Nrf2 expression would prevent cancer cells from protecting themselves from oxidative stress, leading to induction of apoptosis. For example, in a study by Young Cha *et al*., a histone deacetylase inhibitor, valproic acid, was shown to sensitize papillary thyroid cancer cells to the activity of a chemotherapeutic agent, TRAIL, a tumour necrosis factor-related apoptosis-inducing ligand. Consequently, downregulating Nrf2 expression in papillary thyroid cancer cells with upregulated Nrf2 expression led to apoptosis and significantly inhibited tumour growth in an orthotopic thyroid mouse model [43]. So far, there has been successful completion of one clinical trial to date testing the efficacy of SFN as an anti-cancer agent, but no clinical trials have been assigned that test SFN in a combination regimen. In a double-blinded, randomized, placebo-controlled multicenter trial, SFN has shown to significantly reduce levels of prostate-specific antigen (PSA) (44.4% SFN group vs. 71.8% in placebo), an antigen that is commonly elevated in prostate cancer after radical prostatectomy and used as a partial biomarker for “biochemical recurrence”. In addition, the PSA doubling time for the SFN group was 86% longer in comparison to the placebo group, all suggesting that SFN provides clinical efficacy in cancer prevention, specifically for prostate cancer [60].

In another example, metformin, an agent used to treat type 2 diabetes, was found to increase the susceptibility of wild-type p53 breast cancer cell line MCF-7 to TRAIL by upregulating microRNA-34a expression. This upregulation led to the downregulation of Nrf2/Sirt1/Pgc-1alpha pathway and Nrf2 regulated genes (HO-1 and SOD2) that are activated in an antioxidant response. As a result, the breast cancer cells became increasingly susceptible to oxidative stress and hence, TRAIL-induced apoptosis [61]. Nrf2-downregulating agents have displayed propitious outcomes, but there are no clinical trials as of yet to test their anti-cancer capabilities, although these agents warrant further studies.

### Hypoxia and the carbonic anhydrase pathway

**Figure 2:**
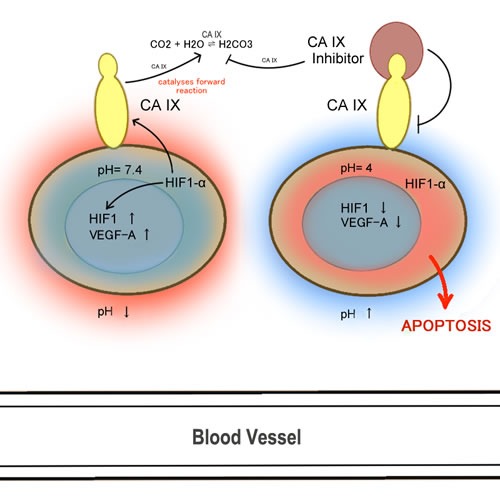
CA IX and angiogenesis

Cancer cells have properties that differ from normal cells, and one of those important properties is their abnormal growth pattern due in part to an erratic vasculature. This abnormal growth pattern consequently results in a large diffusion distance between nutritive blood vessels [62]. The lack of oxygenated blood supplying the tumour creates strong hypoxic conditions favouring the emergence of tumour-initiating cells; the lack of oxygen inhibits tumor differentiation and upregulates programming that allows CSCs to flourish [63]. Additionally, hypoxic conditions drive cancer cells towards adopting anaerobic glycolysis, instead of oxidative phosphorylation, which inherently leads to lactic acid build-up and a lower extracellular pH level in the tumor microenvironment [64]. Moreover, during hypoxia, hypoxic inducible factor-1 (HIF1alpha), a transcription factor, translocates into the nucleus, where it dimerizes with HIF-1beta and leads to the expression of various genes involved in angiogenesis, epithelial-mesenchymal transition, cellular survival, and metastasis. It is interesting to note that regulation of HIF1alpha via prolyl hydroxylase, PHD (the O2 sensor) is another example like Keap1-Nrf2 where the transcription factor HIF1alpha is normally recycled (under normoxia) but is dissociated from PHD under hypoxia. A particularly important gene activated by HIF1alpha is carbonic anhydrase (CA) [65]. CAs help tumours adapt to hypoxic stress by catalyzing the reversible conversion of carbon dioxide to a proton and bicarbonate and thereby neutralizing the acidic conditions of the tumor microenvironment [65, 66]. Although there are a total of 15 CA isoforms in the human body, CAIX [67, 68] and CAXII [69] isoforms have been shown to be the target of many carbonic anhydrase inhibitors (CAIs) because their overexpression in various tumors is correlated with poor survival and progression (Figure 2) [70–72]. CAIX overexpression is correlated with poor prognosis partly because these cells are highly enriched in stemness properties [73–75]; *in vivo* testing has demonstrated that stem cell regulation is highly dependent on pH regulation [76]. Thus, agents that target CA should be considered valuable candidates for cancer research due to their high potential therapeutic benefits.

### Carbonic anhydrase inhibitors in combination therapy

As discussed earlier, carbonic anhydrase inhibitors (CAIs) have multiple treatment purposes, such as in glaucoma therapy, epilepsy, anti-infective treatment, and also certain types of cancers [66, 77, 78]. In the 1970s, CAI was first used for the treatment of peptic ulcers by Ioan Puşcaş and colleagues [79]. The lack of unwanted side effects ultimately resulted in CAI being used for therapies against other medical conditions, and it eventually led to the discovery of CAI being used as an anti-cancer compound [79]. CAIs range from having non-isoform-specific activity to isoform-specific activity, and thus, whether to use pan-CAI treatment verses isoform-specific CAI depends on the type of therapy required [78, 80]. AZ, for example, is a pan-CAI that is currently in the development for the treatment of cancers, although its former FDA-approved use is primarily for glaucoma and epilepsy treatment. AZ has shown promising results in anti-cancer activity when given solely since it displayed anti-invasive effects on renal cell cancer lines and HT29 colorectal cancer cells [81, 82]. However, for the treatment of bronchial carcinoid and bladder cancer, when AZ is combined with SFN, cancer efficacy was additively enhanced compared to monotherapy of each agent; the enhanced efficacy was attributed to increased inhibitory effects on clonogenic and invasive ability *in vitro*, and increased tumor growth inhibitory effects *in vivo* (Figure 1) [15, 28]. SFN has also shown to play a significant role in sensitizing ovarian carcinoma cell line derivatives, adriamycin-resistant A2780/ADR and cisplatin-resistant A2780/CP. Hypoxic conditions provide optimal conditions for tumour growth and SFN modulates the pathways involved in cell proliferation and survival. Specifically, SFN activates various anti-cancer responses such as p53, ARE, IRF-1, Pax-6 and XRE while suppressing proteins involved in tumorigenesis and progression, such as HIF1α, AP-1 and CA IX. SFN has thus shown to reduce chemoresistance and may be a potential agent to be used in conjunction with chemotherapeutics [83]. Recently, benzenesulfonamide derivatives have been synthesized to possess CAI activity for CA I, II and IX while displaying cytotoxic effects on breast cancer cell line MCF-7, with varying IC50s [84]. Another CAIX inhibitor, sulfamate (S4), was tested on a laryngeal tumor model in a study done by Meijer *et al*., but did not display significant anti-tumour and anti-proliferative effects to be used as a single agent [85]. However, in another study, it was postulated that in combination with the proton pump inhibitor lansoprazole it potentiated the effects of melanoma Me30966 cells treated with CAIX specific inhibitor FC9-399A or S4 treatment [86]. This combination resulted in an enhanced dose-dependent tumor inhibitory response and cytotoxicity. Moreover, combination therapy involving CAIs continues to present promising results of enhanced efficacy, such as those shown in a study done by Gieling *et al*., where CAI, AZ, enhanced the efficacy and toxicity of basic toxic agent, doxorubicin (DOX) in human colon carcinoma cell line HT29 by enhancing the cellular uptake of DOX. In addition, CA9/18 cells that have upregulated CAIX expression showed the highest AZ+DOX combinatorial efficacy [87]. Given this growing evidence, it is reasonable to support the use of carbonic anhydrase inhibitors in combination therapies for cancer. Although CAI in combination therapy has shown promising anti-cancer effects in preclinical studies, clinical trials have yet to be conducted. Only one recently completed clinical trial testing the monotherapy treatment of CAI, SLC-0111, for the treatment of solid tumours is reported [88].

### Epigenetics and histone modulation pathway in cancer

Epigenetics is the study of chromatin dynamics with a focus on the regulation of gene expression as opposed to changes in DNA sequences. Epigenetic regulation accounts for the expression and silencing of certain genes and regulates the structure of the genome to produce a cellular homeostatic environment. As a consequence of mutations and stress, the epigenetic modulation pathway can be altered to give rise to cancer and correspondingly up-regulation of proliferative activity. Additionally, cancer cells exhibit a different DNA architecture in comparison to normal cells, as cancer cells are typically less differentiated and the chromatin contains more histones that compact the DNA [89]. As a result, cancer cells typically expose fewer open regions of DNA for transcription. As cancer cells become characteristically less differentiated, they behave more invasively because differentiation inhibits cellular movement [90]. Consequently, epigenetic regulation that modulates gene expression becomes highly active in cancer and therefore offers valuable therapeutic targets.

There are various ways the epigenome is modified, including DNA methylation, histone acetylation, histone phosphorylation, histone ubiquitination, and other post-translational modifications [91]. Mutations in the enzymes that regulate the epigenome can potentially lead to cancer-like states because of their ability to create gross effects [92]. Cancer mutations, such as mutations in histone acetylase, histone deacetylase (HDAC), and transcription factors, can lead to either an upregulation or downregulation of gene products they target and therefore the ability to drive a tumour into a more stem-like, undifferentiated state [92]. Due to the properties of epigenetic modifiers, many important cancer treatments target them.

**Figure 3:**
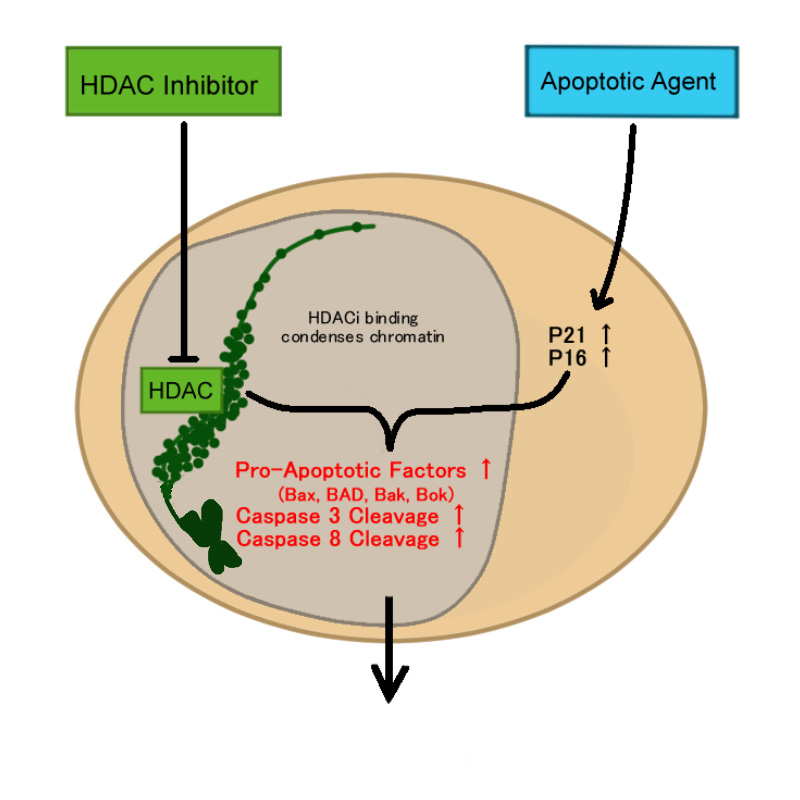
HDAC and apoptosis

In the past 10 years, histone deacetylase inhibitors (HDACi) in particular, have emerged as important agents of interest in clinical trials for all types of diseases [93]. The specific appeal of HDACi for cancer treatment is due to their vast array of anti-cancer effects, such as the ability to induce differentiation, cell cycle arrest, apoptosis and inhibit angiogenesis [94]. HDAC itself has also exhibited extensive activity in cancer cells [94] because HDAC aids in cancer morphology by compacting chromatin, creating an un-differentiated state. In addition, tumour cells are more sensitive to HDACi-induced apoptosis in comparison to normal cells, albeit the reason is still unknown [94]. Moreover, the specific function of HDACi is to inhibit the activity of histone deacetylases, enzymes that deacetylate the lysine residues on histone molecules, leading to the inaccessibility of transcriptionally active regions of DNA (Figure 3). HDAC plays a role in epigenetics and chromatin dynamics by essentially regulating gene expression.

Although under-regulation of HDACs has been involved or present in many cancers, over-regulation of HDACs has been found in a variety of cancers. For example, HDAC10 (one class of HDAC) have shown to be increased in gastric cancers [95]. In another study, it was found that HDAC1 mRNA expression was upregulated in non-small cell lung cancer and was associated with a low differentiated grade tumour, while lung adenocarcinoma with upregulated HDAC1 mRNA expression was associated with poor prognosis [96]. Also, a common modification of HDAC that leads to many human cancers is a global loss o’{f monoacetylation of the Lys16 residue of histone H4 [97]. Consequently, HDACi are able to effectively inhibit the invasiveness and aggressiveness of cancer phenotypes that express this Lys16 mutation amongst other HDAC mutations [97].

### Histone deacetylase inhibitors in combination therapy

Research on creating novel therapeutic treatments utilizes the knowledge of common epigenetic modifier mutations in specific cancers and uses them as therapeutic targets. For example, studies have introduced epigenetic modifier gene mutations into mouse hematopoietic stem cells. Results show that the epigenetic mutation increased cancer stem cell properties such as self-renewal capability [98]. Furthermore, therapeutic treatments, such as HDACi targeting epigenetic modifiers, are emerging primarily because of the plasticity of the epigenome and its reversibility. HDACs are common targets for cancer therapy as their dysregulation is associated with many forms of cancers; HDACi is therefore frequently tested in clinical trials. Trichostatin A was the first HDACi tested in clinical trials while vorinostat, a potent inhibitor of HDAC1, HDAC2, HDAC3 and HDAC6, was the first FDA-approved HDACi for the treatment of persistent, progressive or recurrent cutaneous T-cell lymphoma [99, 100]. Due to the successful results of vorinostat, many other HDACi has been applied in the treatment of cancers, such as romidepsin for cutaneous T-cell lymphoma, and Panobinostat for the treatment of multiple myeloma [101, 102].

Repurposed HDACi have also been tailored for the treatment of diverse cancers, such as valproic acid (VPA) originally used for the treatment of epilepsy, and romidepsin originally FDA-approved for hematological cancers such as cutaneous and peripheral T-cell lymphoma but have also shown anti-cancer effects in solid tumours [103, 104]. Additionally, repurposed HDACi in combination with other agents have been tested in clinical trials; however, not all displayed promising results due to the build-up of toxic side effects or insignificant effects. For instance, recently a phase II randomized study was done testing the effects of VPA and decitabine on patients with acute myelogenous leukemia and myelodysplastic syndrome [105]. The results demonstrated that there were no significant differences between the effects of single agent treatment with decitabine and combination therapy with decitabine and VPA. Complete remissions of 31% and 37% (p=0.497) respectively were observed, although preclinical studies demonstrated a significant difference in growth inhibition and apoptosis. Similar results were obtained in a randomized phase III study of VPA in combination with an all-trans retinoid for intensive therapy in the treatment of acute myeloid leukemia in the elderly, where the combination resulted in low complete remission rates and increased VPA-related hematological toxicities [106]. The combination of VPA (75-100 mcg/mL) and 5-azacytidine (5-AZA) (75 mg/m^2) delivered daily for 10 days in patients with advanced cancers such as colorectal, melanoma and breast cancers, were deemed safe, but did not particularly result in enhanced anti-cancer efficacy compared to 5-AZA monotherapy [107]. Although these studies do not particularly demonstrate the advantages of incorporating VPA, further studies of VPA in combination with other cytotoxic agents are still warranted. It may be that VPA is too weak of an HDACi as compared to others to be discussed.

Romidepsin, another repurposed agent, was tested in combination with gemcitabine on solid advanced tumours of the pancreas, breast, non-small cell lung cancer, ovaries, and others [103]. Results have shown a partial response of 7% of patients while 52% of patients responded with a stable disease. In addition, a majority of the adverse events seen were non-hematological, such as nausea, vomiting, and fatigue while 47% of patients also experienced hematological abnormalities. As stated in the study, a phase 2 trial is warranted and demonstrates the promising effects of repurposed drugs in combination therapy. Moreover, FDA-approved HDACi were further assessed in combination with other therapeutic agents in clinical trials. For example, in a phase 1 clinical trial study, proteasome inhibitor marizomib (0.7 mg/m^2 on days 1, 8 and 15-82) in combination with vorinostat (300 mg/day on days 1-16) displayed enhanced therapeutic benefits compared to either agent alone in patients with melanoma [108]. The combination of both agents did not increase toxicity beyond the toxicity profile of each agent alone and appeared to synergistically induce cellular stress levels because marizomib inhibits proapoptotic factor degradation while vorinostat inhibits the formation of aggresomes that marizomib induces. As a result, tumour size decreased in in 39% of patients.

There is currently a synthetic benzamide HDACi, MS-275 (entinostat or SNDX-275) that has undergone several clinical trials [109, 110]. MS-275 selectively inhibits HDAC1/2 [111]. Since HDAC1 expression level is upregulated in prostate, gastric, colon and breast carcinomas whereas HDAC2 expression level is upregulated in colorectal, cervical and gastric cancers, MS-275 treatment on these types of cancers seem promising [112]. This therapeutic agent does not cause cardiotoxicity, a major benefit of HDACi, as reported in phase 1 clinical trials on solid and hematologic malignancies [109, 113]. However, its half-maximal inhibitory concentration is higher than most hydroxamic acid HDACi, being in the micromolar range as opposed to nanomolar [93]. Due to MS-275 requiring high inhibitory concentrations to give the desired effect, it can be argued that MS-275 in combination with another therapeutic agent might demonstrate potentiation effects, and thus lower the therapeutically required dose of MS-275. Notably, in our preclinical study, we discovered that the combination of AZ (40 μM) and MS-275 (0.75 μM) displayed significantly higher effects on cell viability, reduction in migration capacity, and growth inhibitory effects compared to either agent alone for the treatment of neuroblastoma. Although MS-275 is a potent HDACi, it is toxic to both normal cells and cancer cells. In our study, we saw that AZ potentiated the efficacy of MS-275 at a lower dose, which creates the highest efficacy required with the lowest possible toxic effects on normal cells [114]. This finding is important since both drugs used in our study are repurposed FDA-approved drugs and the doses fall within the clinically accepted range. Combination therapy that includes MS-275 has also undergone clinical investigation. For example, in a phase I/II study, the combination of DNA methyltransferase inhibitor, 5-azacytidine (30 mg/m^2/d, 40 mg/m^2/d) and MS-275 (7 mg) was administered to patients with refractory advanced non-small cell lung cancer. Results obtained were encouraging and warranted further study. This combination was safe and well-tolerated, and the combination therapy was shown to improve overall survival rate (6.4 months vs. 4 months) and progression-free survival compared to the current therapeutics available for this type of cancer [115, 116]. In summary, HDAC poses a promising target in the development of therapeutic treatments and repurposed HDACi warrant further clinical studies.

### Autocrine growth factors in cancer survival

Cellular physiology relies on cell signaling for homeostatic equilibrium and communication. The secreting cell varies in the type of secretion effector method with regards to signaling. For instance, the secreting cell can secrete substances either in an endocrine, paracrine, or an autocrine fashion. When the effector substance is secreted into the blood stream affecting a remote cell, it is known as endocrine communication. The paracrine and autocrine forms of communication are defined as the secretion of messenger substances from one cell to a neighboring cell through diffusion, and from one cell onto itself through ligand/receptor interaction, respectively [117]. One type of effector substance that is prevalent in cancers operating to enhance a malignant characteristic is the autocrine growth factor where pro-proliferative activity is mediated through an autocrine growth loop [117]. There are many types of autocrine growth factors, with the major ones in cancer being vascular endothelial growth factor (VEGF), epidermal growth factor (EGF), insulin-like growth factor-2 (IGF-2), epidermal growth factor receptor (EGFR) and 5-hydroxytryptamine (5-HT; serotonin), to name a few [117–120]. If growth factors do not respond to negative regulatory signals, cancer may result as a consequence of excessive proliferation. VEGF acts by inducing angiogenesis in hypoxic conditions [119] and will be discussed in depth in the next section (Figure 2). IGF-1 is a growth factor involved in cancer proliferation and inhibition of apoptosis. IGF-1 mechanism of action involves the binding of IGF-1 to the IGF receptors, which correspondingly activates intrinsic tyrosine kinase activity, leading to autophosphorylation and subsequent secondary messenger effects [121]. According to some epidemiologic studies, patients with type 2 diabetes are at a higher risk of developing cancer due to the elevated activity of IGF-1[122].

5-HT, a potent bioactive molecule that primarily acts on G-coupled protein receptors/5-HT receptors, has diverse effects in the body, ranging from its neurotransmitter role in depression to its hormonal role in cancer [123]. There are 14 different 5-HT receptors, which are distinguished based on their location on the axonal terminal. Additionally, whether the signaling is excitatory and inhibitory depends on the type of 5-HT receptor on the membrane [123]. 5-HT biology is an important area of cancer research as it has presented a significant involvement in cancer. Specifically, 5-HT has displayed tumorigenic effects in various cancers in terms of cancer proliferation, anti-apoptotic effects, angiogenesis, and cellular survival, through the activation of a second messenger cascade [124, 125]. For instance, 5-HT receptors were shown to be overexpressed in bladder cancer [126], prostate cancer [127], breast cancer [128], and neuroendocrine type cancers [118]. Recently, the role of 5-HT attenuation on angiogenesis has been discovered, as it has been shown that 5-HT stimulates angiogenic signaling kinases, which also demonstrates its role in metastasis [129]. Our lab's recent focus on the role of 5-HT in cancer has yielded several reports, illustrating the significance of 5-HT as an autocrine growth factor in cancer and specifically, the pathobiology of respiratory cancers [15, 130–132]. In normal lung tissue, neuroepithelial bodies (NEB) produce and secrete significant amounts of 5-HT especially under hypoxic conditions. HIF1alpha has been shown to be upregulated in a significant portion of 5-HT positive cells [130, 131] making a connection with this regulatory pathway. It was discovered that hypoxia induces the release of 5-HT from neuroepithelial bodies [133]. As the studies also used 5-HT-producing lung tumour cell lines, 5-HT is suggested to serve as an autocrine factor regulating the proliferation of the cells through an autocrine growth factor effect. Furthermore, in the neuroendocrine bronchial atypical carcinoid cell line H720, 5-HT appeared to stimulate pro-proliferative effects via 5-HT2 and 5-HT3 receptors [118]. Thus, among the other growth factors, 5-HT has displayed vigorous proliferative roles in cancer and as a consequence, 5-HT may be vulnerable to therapeutic intervention if the malignancy is dependent on an autocrine growth factor loop [117].

### Targeting 5-HT autocrine growth factor in combination therapy

The 5-HT autocrine growth factor receptor is a promising target for therapeutic intervention for cancer cells that overexpress 5-HT receptors in *in vitro* and *in vivo* studies. Specifically, in neuroendocrine tumors, it was discovered that 5-HT_2B_ receptor was only expressed in neoplastic small intestinal neuroendocrine tumors of enterochromaffin (SI-NET EC cell-derived) cell line and not in normal EC cells [134]. Proliferative activity was reduced in the KRJ-I cell line of SI-NET when targeted by PRX-08066, a 5-HT_2B_ inhibitor, through decreased 5-HT autocrine secretion [134]. Thus far, 5-HT targeting agents in combination therapy have yet to be tested in clinical trials for cancer therapy but have displayed significant outcomes in preclinical settings. For instance, it has been previously reported that repurposed acetazolamide (AZ) reduced 5-HT secretion and proliferation in human renal carcinoma cells [81, 135, 136]. Additionally, it was reported that SFN downregulates 5-HT receptor expression in Caco-2 cells [137] Conclusively, in our previous study, the combination of AZ and SFN significantly reduced the 5-HT-induced growth of bronchial carcinoid cell lines H727 and H720 in an additive manner [15]. Due to the role of 5-HT in proliferation, tumorgenicity, and in carcinoid syndrome, as it was discovered that carcinoid syndrome is due to an overexpression of 5-HT secretion [138], it would be beneficial to consider clinical trial testing of the combination of cytotoxic agents and therapeutic agents that target 5-HT directly or indirectly, in order to potentially create either a synergistic or additive effect for efficacy.

### Targeting other autocrine growth factors in combination therapy

There currently are various therapeutics in the pharmaceutical drug market that target autocrine growth factors in cancer. EGFR, a growth factor receptor that is often regulated through the autocrine growth factor loop in normal bronchial epithelial cells and lung cancer cells [120], was investigated in a phase II study by Ichihara *et al*., where gefitinib in combination with bevacizumab was administered as a potential first-line therapy for patients with non-small-cell-lung cancer with EGFR mutations. This combination resulted in a 69% partial response, 4.7% complete response and 23.8% stable disease; adverse effects were tolerable. The results conclusively warrant additional studies [139]. Figitumumab, a monoclonal antibody targeting IGF1R, combined with chemotherapeutics displayed varying results. For example, in a phase Ib dose-escalation open-label study, figitumumab (20 mg/kg) combined with docetaxel (75 mg/kg) was deemed safe in patients with advanced malignancies such as sarcoma, non-small cell lung cancer, gastric, and ovarian cancers [140]. However, in a phase II randomized study of figitumumab and docetaxel combination on patients with metastatic castration-resistant prostate cancer, serious toxic adverse effects, including grade 3/4-5 events, were observed, rendering this combination unviable. [141]. In a randomized phase III trial, the addition of IGF1R inhibitor, ganitumab, displayed insufficient efficacy as a first-line therapy for the treatment of patients with adenocarcinoma of the pancreas, as it did not result in better survival rates compared to gemcitabine monotherapy [142]. However, in a phase 2 randomized double-blind study, ganitumab in combination with gemcitabine displayed a better 6-month survival rate and overall survival rate compared to the placebo in patients with metastatic pancreatic cancer, which demonstrates that IGF1R inhibitors may display propitious outcomes in combination therapy if the right combinations are used [143].

### Angiogenesis- a cancer-sustaining pathway

Neoplasms that become aggressive are characterized as malignant and have the potential to metastasize. In the malignant state, tumour cells can potentially separate from the primary mass and migrate towards capillaries, eventually invading through the endothelium and entering the blood circulation. Thereafter, tumour cells can circulate throughout the body and migrate out of the capillaries into distant organs, where they can form another tumour, a process termed metastasis. Once cancer has metastasized, it becomes very difficult to treat by conventional therapeutics [144]. Therefore, it becomes important to find pharmaceutical agents that prevent metastasis from occurring and one of the approaches is by inhibiting angiogenesis. Due to the high metabolic requirements of cancer cells for growth, and the increased diffusion distance between cancer cells and blood vessels, tumours generally experience hypoxic conditions. As a result, angiogenesis helps to sustain their metabolic needs but also induces their invasive characteristics. This process is firstly initiated by hypoxia-induced HIF1alpha expression. HIF1alpha has been shown to mediate the upregulation of vascular endothelial growth factor (VEGF) [145]. HIF1alpha binds to promoter elements in the transcriptional region of VEGF-A, an autocrine growth factor that contributes to the process of angiogenesis, and thereby induces its expression [146]. Moreover, there are a family of ligands (VEGF-A to-D) that bind to the vascular endothelial growth factor tyrosine kinase receptor superfamily, VEGFR-1, and VEGFR-2; however, VEGF-A and –B have shown to have the highest affinity for these receptors [147]. VEGF-A bound to VEGFR-2 produces the most prominent angiogenic effects [147]. There are many cancers that exhibit high angiogenesis activity either through overexpressing VEGF or its receptors or downstream effectors in the VEGF/VEGF receptor pathway, as in breast cancer [148], invasive bladder cancer [149], colon cancer, [150] and melanoma [151]. Thus, anti-angiogenesis therapy would be an appropriate approach as an anti-cancer modality.

Mechanistically, drugs that inhibit angiogenesis deprive tumour cells of vital nutrients and oxygen, and therefore they suppress tumor growth and progression [152]. Anti-angiogenic activity is evident in metronomic chemotherapy, a new approach to controlling tumor growth. Here, chronic administration of a low dose of drugs with anti-angiogenic properties is an effective strategy in cancer treatment [153, 154]. As a result, it is important to target factors upregulated in hypoxic conditions prior to metastasis, such as VEGF/VEGFR for the achievement of a potent anti-cancer effect.

### Anti-angiogenesis therapeutic agents and their use in combination therapy

Vascular endothelial growth factor (VEGF) and its receptor, vascular endothelial growth factor receptor (VEGFR) are normally expressed in many cells but show significant upregulation in many types of cancers, including bladder carcinoma [155], and breast angiosarcoma [156]. As a result, many therapeutic agents are aimed at targeting the VEGF/VEGFR complex. Recently, a repurposed anti-angiogenic agent has been selected and tested in clinical trials. Itraconazole, an anti-fungal agent, has shown to inhibit endothelial cell proliferation and migration, and inhibit activation of VEGFR2 and FGFR3 *in vitro* and *in vivo* in non-small cell lung cancer cell lines (NCI-H358, NCI-H1838, NCI-H596 and NCI-H1975) [157]. Furthermore, itraconazole was used in combination with pemetrexed, a chemotherapy drug, for the treatment of non-squamous non-small-cell lung cancer in a phase II clinical trial. Results demonstrated significant differences in the overall survival media of 8 months and 32 months between the control, pemetrexed monotherapy, and experimental, pemetrexed and itraconazole combined treatment. In addition, toxicity profiles were not significantly different between the two treatments [158]. Furthermore, the combination of itraconazole and chemotherapy agents (irinotecan-based) on metastatic pancreatic cancer showed promising results (8% complete response, 39% partial response, 32% stable disease, 13% progressive disease and 47% response rate) with limited toxicities [159].

One of the more common anti-angiogenic agents is bevacizumab, an antibody that displays VEGF inhibitory activity through antibody mediation. In a study by Yue *et al*., bevacizumab in combination with turmeric ethanolic extract (with absorbable curcumin) exhibited enhanced anti-cancer effects in HT29 colonic cancer cells compared to monotherapy through tumour growth inhibition, pro-apoptotic effects and blood vessel growth inhibition [160]. In a phase 2 open-labeled randomized trial, erlotinib combined with bevacizumab was used as a first-line therapy for patients with advanced non-squamous non-small-cell lung cancer with EGFR mutations [161]. This combination has shown promising results in terms of anti-cancer efficacy as all patients’ tumours were reduced in size, while progression-free survival was maintained and 69% of patients displayed an objective response compared to 64% of patients in the erlotinib alone group. While this combination displayed some toxic effects, efficacy and safety profiles are still being elucidated through BELIEF (NCT01562028) and ACCRU RC1126 (NCT01532089).

Furthermore, adaptive resistance may pose limitations on single agent treatment with anti-angiogenic drugs. Adaptive resistance occurs when the treatment creates unforeseen changes in the tumour morphology and vascularization, and this resistance is partly due to the induction of hypoxic factors that recruit other pro-angiogenic factors not targeted by the original therapeutic intervention [162]. However, adaptive resistance can be prevented by the use of combination treatment, such as shown in a study by Hartwich *et al*., where the suppression of HIF1alpha using topotecan potentiated the effects of bevacizumab in neuroblastoma NB1691 cell line, significantly reducing tumour growth compared to either treatment alone [163]. The additional targeting of hypoxia-inducing factors such as HIF1alpha or carbonic anhydrases may contribute to enhanced anti-tumour activity and reduce angiogenic resistance since it would target a different angiogenic pathway that contributes to the expression of VEGF and blood vessel formation. In a preclinical study, we demonstrated that the combination of the anti-angiogenic agent pazopanib and a topoisomerase inhibitor, oral metronomic topotecan, inhibited tumour growth and reduced microvessel formation in neuroblastoma xenografts, although some resistance was observed [31]. Due to this promising result, our study recently received permission to continue in a clinical trial setting (ClinicalTrials.gov identifier: NCT02303028). Avastin, an anti-angiogenic drug, may also potentiate chemotherapeutic drugs and consequently sensitize cancer cells to the cytotoxic effects of chemotherapy [164]. First, metronomic therapy acts as anti-angiogenic therapy, inducing hypoxic conditions as a result of lack of blood flow to the tumour. Hypoxic conditions stabilize HIF1alpha, trans-activating genes such as VEGF. However, avastin specifically inhibits VEGF, and therefore this combination demonstrates that avastin sensitizes cancer cells to the cytotoxic effects of metronomic chemotherapy [164].

These aforementioned studies demonstrate the effectiveness of repurposed and regular anti-angiogenic agents combined with factors that decrease or inhibit the hypoxic conditions or factors of the tumor microenvironment. Thus there is a need for more combination interventions that target these pathways either synergistically, additively, or in a potentiating manner.

### Apoptosis pathway in cancer

Apoptosis, or programmed cell death, is a vital function during normal development, as cells that have undergone irreversible DNA damage or have been negatively affected by carcinogens can lead to tumour formation and potentially cancer. During cancer development, inhibition of apoptosis is complemented by activation of pro-survival mechanisms for cancer cell progression and growth. Thus, many therapeutic agents are designed to target apoptotic and cell cycle pathways. There are two main apoptotic pathways involved in normal human physiology: the extrinsic pathway and the intrinsic pathway. The extrinsic pathway constitutes death receptors and ligands, FasL/FasR, APO-1/Fas (CD95), TNF-alpha/TNFR1, Apo3L/DR3, Apo2L/DR4, and Apo2L/DR5 that are part of the tumour necrosis factor (TNF) gene superfamily. These death receptors trigger intracellular signaling that results in the cleaving and activation of caspases such as caspase-3 and caspase-8, which ultimately lead to apoptosis [165, 166]. The CD95 and TNF-related apoptosis-inducing ligand-receptor 1/2 are the most commonly targeted death receptors in therapeutic intervention. CD95 is a receptor-regulated in immune activity, where the CD95 ligand activates T-cells, producing an immune response that leads to programmed cell death [165]. Cancer cells have evolved mechanisms that disrupt the normal physiological response of apoptosis through either downregulating the expression of surface death receptors, such as CD95 and TRAIL receptors, or by overexpressing decoy receptors that are involved in preventing the normal apoptotic response [165]. This apoptotic disruption is strongly correlated with drug resistance; for example, downregulation of CD95 surface expression leads to therapeutic treatment resistance in leukemia [167].

An equally important apoptotic pathway that cancer cells can manipulate is the intrinsic pathway of apoptosis. Members of the Bcl family, which are pro-apoptotic or anti-apoptotic factors, permeabilize the outer mitochondrial membrane [165]. This process leads to the release of pro-apoptotic proteins from the intermembrane space into the cytosol, leading to apoptosis activation [165]. In a study by Cotter *et al.,* it was postulated that various cancers have elevated levels of anti-apoptotic Bcl-2 proteins [168]. Bcl-2 proteins contribute to cell survival in a two-fold manner: Bcl-2 inhibits adaptors that are required for apoptotic activation and also cleaves caspases, leading to the nuclear and cell fragmentation that are characteristic of apoptosis [169]. Bcl-2 is used as a prognosis marker in non-small-cell lung cancer (NCI-H460), and was correlated with unfavourable histology in neuroblastoma (stages I-IV) and was overexpressed in prostate cancer (LNCaP) [170–172]. Thus, it can be argued that therapeutics that aim to target these anti-apoptotic or pro-survival proteins would lead to increased anti-cancer efficacy.

### Apoptosis targeting agents in combination therapy

Many therapeutic agents aim to target factors that either upregulate pro-apoptotic factors or inhibit pro-survival mechanisms (Figure 3). Repurposed therapeutic agents have demonstrated many advantages both therapeutically, temporally, and financially but there are only a couple of repurposed drugs with apoptosis-inducing properties that are currently under clinical investigation. One repurposed agent that possesses the ability to induce apoptosis indirectly is nitroglycerin, an agent used to treat angina. Nitroglycerin is a nitric oxide (NO) donor, and nitric oxide has been found to induce apoptosis as a secondary effect alongside its primary role in downregulating HIF1alpha and inhibiting angiogenesis [173, 174]. Furthermore, one randomized phase II trial demonstrated that nitroglycerin in combination with the chemotherapeutics vinorelbine and cisplatin improved overall survival of patients with untreated stage IIIB/IV non-squamous cell lung cancer [175]. Due to the ability of nitroglycerin to ameliorate hypoxic conditions, it was postulated that it might improve resistance when combined with chemotherapy, although definite answers have not yet been provided. The results of this study demonstrated that nitroglycerin improved the response rate to vinorelbine with tolerable toxicity, and also stated that a phase III trial is warranted, although one has not yet been initiated.

An additional repurposed therapeutic agent that has gained some attention from researchers is clarithromycin (CAM), a macrolide antibiotic normally used to treat bacterial infections involving the skin and respiratory system [176]. This agent has been shown to enhance endoplasmic reticulum stress-mediated apoptosis when combined with bortezomib, a proteasome inhibitor, although CAM has shown to only be efficacious when administered in a combination regimen in breast cancer and myeloma cells [177, 178]. In another study, the combination of HDACi SAHA, with bortezomib and CAM, induced apoptosis in breast cancer cells. This particular combination was chosen because these compounds work synergistically and inhibit the ability of cancer cells to adapt to cellular stress [179]. Although *in vitro* studies using CAM in combination with other agents have shown promising results, *in vivo* and clinical testing have not yet been assessed; further studies are recommended.

There are still many apoptosis-inducing agents out in the market that are being evaluated in clinical trials, whether as a single agent or in combination therapy [180–182]. For instance, in a randomized phase II study, 10 mg/kg of conatumumab, a monoclonal antibody agonist against TRAIL, combined with FOLFIRI chemotherapy (folinic acid, fluorouracil, and irinotecan) displayed tolerable toxicity profiles and modest progression-free survival in mutant KRAS metastatic colorectal cancer [183]. Although this combination was not statistically significant enough for further investigation, clinical investigations of other combination therapies involving conatumumab may still provide efficacious results. Nonetheless, studies displaying the efficacy of combination therapy set a precedent for more research combining therapeutic agents that induce apoptosis.

## DISCUSSION

Multiple therapeutic designs have been developed to target various cancer pathways. Combination therapy has provided the most effective results with regards to anti-cancer effects. Its superiority stems from the ability to target multiple pathways, which essentially minimizes drug resistance because cancer cells are frequently incapable of adapting to the simultaneous toxic effects of two therapeutic agents [184]. Moreover, various pathways are dysregulated in cancer cells and have disrupted homeostatic environments that generally contribute to the rapid proliferation rate. For instance, studies have shown that various cancers have mutations in tumour suppressor genes, such as p53, that normally function to activate cell cycle arrest when DNA is damaged. However, if tumour suppressor genes are mutated, accumulation of damaged DNA and inhibition of cell cycle arrest contribute to increasingly rapid proliferation rates and a more aggressive cancer [185]. Additionally, in cancer cells, upregulated production of autocrine growth factors or an upregulated autocrine loop can further contribute to the growth of tumour cells [186, 187]. If the tumour size has increased substantially, neoadjuvant chemotherapy might be indicated prior to surgical resection of the tumour; here, combination chemotherapy may potentially aid in survival. With regards to autocrine growth factors, if VEGF is upregulated, metastasis can also occur, which may worsen the prognosis and survival rate [188, 189]. Therefore, targeting various pathways via multiple drug combinations can increase the chance of disease control and decrease the chance of cancer cells becoming increasingly malignant and incurable. Also, in some cases, the dose requirement of each agent in combination therapy can be reduced, which reduces the side-effects compared to monotherapy, albeit some combination therapies have been shown to increase toxicity [190, 191]. An additional advantage of combination therapy is that multiple drugs can target the heterogeneous nature of tumours, correspondingly increasing the chance of killing all cancer cells, including the cancer stem cell population that is known to contribute to drug resistance and cancer recurrence after remission in later years [192–194].

It should be noted, however, that studies have also revealed some disadvantages of combination therapy use in cancer. Firstly, the combination of multiple agents can synergistically or additively create therapeutic benefits, but can equally produce unwanted side-effects [191]. This can make it difficult to identify the responsible agent and as a result, it would be difficult to assess which agent's dose should be lowered. If the therapeutic agents act similarly, where their side-effect profiles are similar, the accumulation of side-effects can create more severe clinical symptoms and grade 3/4 toxicities that can severely impact and alter the patient's life expectancy. As an example, in a phase I/II study performed by Berdeja *et al,* the combination of panobinostat and carfilzomib resulted in treatment-related heart failure (2%), while treatment-related death rose (2%) in patients with relapsed/refractory multiple myeloma [195]. Obviously, complications from combination therapies can create further problems for patients in terms of financial and general well-being. Drug interaction should be thoroughly considered when creating a combination therapy regimen for cancer [196]. For instance, one drug may inhibit the metabolic activity of the secondary or tertiary agent, formally leading to the buildup of toxicity which consequently has a detrimental effect on the patient's health.

Combination therapies that include one or more anti-cancer agents that target the pathways discussed above, such as HDACi, CAi, 5-HT receptor inhibitors, anti-angiogenic drugs, and pharmaceutical agents targeting antioxidant pathways may display promising results in cancer research. This is evident as the PubMed database has displayed many results when using the keywords: histone deacetylase inhibitors, HDACi, carbonic anhydrase inhibitors, 5-HT receptor inhibitors, 5-HT inducers, 5-HT agents, autocrine growth factor inhibitors, anti-angiogenics, Nrf2 inducers and Nrf2 inhibitors. It is particularly important to understand the interactions between two or more anti-cancer agents in a combination regimen to create the maximum efficacy with the least amount of toxicity. There are many possible agents that can combine to potentially result in significant therapeutic efficacy, but that have not yet entered into full investigation. For example, although bevacizumab has shown promising anti-angiogenic activity, it does not inhibit the interaction between VEGF RTKs and neuropilin-1 (NRPs), which are contributing factors to cancer stem cell survival [197]. VEGF/NRP cellular signaling has also shown to contribute to tumour cell survival by inducing the PI3K-AKT pathway, an important pathway in tumour cell survival [198]. There have been limited studies investigating the combination of agents that target both of these angiogenic factors. With such knowledge, it is reasonable to search for a combination therapy regimen that includes the targeting of VEGF and NRP interaction, while simultaneously displaying anti-VEGF activity in order to contribute to the most promising result.

## CONCLUSION

Our review demonstrates that therapeutic agents currently on the market are being used in combination therapies that have either completed their clinical evaluations, are still in progress, or warrant prospective clinical trials. In terms of economic benefits, repurposed therapeutic agents tailored to cancer treatment have demonstrated promising results in the clinical setting and have gained attention from oncologists worldwide... Combination therapies that involve a repurposed therapeutic agent have also demonstrated enhanced efficacy and efficiency. Notably, drug repositioning passes toxicity and safety profiles, and when used in combination treatments shows enhanced efficacy in an additive or synergistic manner, potentially also reducing drug resistance.
